# Preliminary usability evaluation of a teleoperated humanoid robot for pediatric waiting-room observation: clinical and technical professionals’ perspectives

**DOI:** 10.3389/frobt.2026.1943197

**Published:** 2026-09-18

**Authors:** Antea Scrocco, Samuele Bordini, Riccardo Kristen Simi, Elisa Matteucci, Elena Beani, Giovanni Cioni, Eugenia Conti, Maria Chiara Di Lieto, Giorgio Grioli, Antonio Bicchi, Giuseppina Sgandurra, Manuel Giuseppe Catalano

**Affiliations:** 1 Department of Developmental Neuroscience, IRCCS Stella Maris Foundation, Pisa, Italy; 2 Department of Neuroscience, Psychology, Medication Area and Child Health (NEUROFARBA), University of Florence, Florence, Italy; 3 Centro Di Ricerca E. Piaggio E Dipartimento Di Ingegneria Dell’Informazione, University of Pisa, Pisa, Italy; 4 Soft Robotics for Human Cooperation and Rehabilitation, Istituto Italiano di Tecnologia, Genova, Italy; 5 Department of Clinical and Experimental Medicine, University of Pisa, Pisa, Italy; 6 Physical AI Technologies for Human-Robot Coevolution, Istituto Italiano di Tecnologia, Bergamo, Italy

**Keywords:** assistive robotics, humanoid, pediatric healthcare, system usability, telepresence avatar

## Abstract

The field of assistive and social robotics is increasingly being applied within pediatric medicine, where waiting rooms provide an interesting setting for conducting ecological behavioral observations. Yet, there is a dearth of studies investigating teleoperated humanoid robots in their capacity as tools for naturalistic observation in an unstructured environment, a use that requires a very high level of user-friendliness of the robot for non-specialist users. In this study, the humanoid robot Alter-Ego was evaluated as a telepresence avatar to support remote observation in the waiting room of a child neuropsychiatry clinic. As a preliminary step toward future clinical evaluation, the study was conducted without the direct involvement of pediatric patients and focused on the usability, perceived cognitive workload, and acceptability of the platform among 13 professionals with clinical and technical backgrounds. The results demonstrated acceptable system usability, with an overall mean System Usability Scale score of 71.3. Regarding cognitive workload, the NASA-TLX identified Mental Demand and Effort as the most demanding dimensions, while Frustration levels remained low. The Godspeed Questionnaire revealed a highly positive perception of the platform’s Animacy and Likeability, alongside good scores for Perceived Safety and Perceived Intelligence. In exploratory correlation analyses, no significant correlations were found between prior virtual reality (VR) or gaming experience and any of the outcome measures considered. In conclusion, the findings suggest that the Alter-Ego platform may be a usable and acceptable teleoperated tool for clinical and technical professionals, providing a basis for future evaluation in pediatric clinical settings.

## 1 Introduction

In recent years, robots have increasingly shared environments with their human counterparts, ranging from industrial settings (Liu et al., 2024) to healthcare facilities (Okamura et al., 2010; Silvera-Tawil, 2024). This transition is facilitated by the development of more intelligent and intrinsically safe robotic systems, alongside intuitive Human-Robot Interfaces that enable teleoperation even for non-expert users. Among the emerging solutions in these contexts, humanoid platforms are gaining significant traction (Siciliano and Khatib, 2020, 1–6; Mukherjee et al., 2022). Their anthropomorphic design allows them to seamlessly navigate unstructured environments built for humans, while providing an intuitive kinematic mapping between the operator and the robot body, thereby streamlining remote teleoperation.

Beyond their assistive roles, social robots function as “relational artifacts,' eliciting social responses that positively influence patient compliance and emotional wellbeing (González-González et al., 2021; Ragno et al., 2023; Heerink et al., 2016). These devices effectively bridge the gap between functional medical tools and human interaction.

The inherent social appeal of humanoid robots has proven beneficial in direct healthcare; for instance, they have been successfully employed to improve the mental wellbeing of elderly patients (Bhandari and Baral Sapkota, 2023). In pediatric contexts, social robots have been shown to increase positive emotions and engagement (Jeong et al., 2018), reduce stress (Beran et al., 2013; Liverpool et al., 2020; Trost et al., 2019), and serve as effective distractors capable of significantly reducing children’s pain and anxiety during medical procedures (Jeong et al., 2015). Consequently, in recent years, the use of social robots with children has become increasingly widespread, sparking growing interest within the scientific community regarding their potential to improve children’s experiences in healthcare settings. Furthermore, it has been shown that the presence of a robot can facilitate the bond between the child, the healthcare provider, and the caregiver (Neerincx et al., 2021), also helping to reduce anxiety and increase satisfaction with the service inside the emergency room waiting area (Sivakumar et al., 2021). Waiting rooms prior to pediatric appointments offer a unique, spontaneous, and less structured setting for observing children’s behavior and development compared to traditional clinical examination rooms. Here, children can interact with and play alongside their peers, freely expressing their emotions and behaviors that might not emerge during more structured clinical evaluations (McMorran-Young et al., 2021). Hence, deploying a robotic platform in this scenario may provide healthcare professionals with an additional tool for remotely observing interactions and behaviors in a less structured environment. Most studies in the literature on the use of robots in hospital waiting rooms have focused on the general pediatric population; however, it is particularly important to explore the application of this innovative technology within the child neuropsychiatry department, where, given the extreme heterogeneity of patients with neurodevelopmental disorders and disabilities, the challenges are even more complex (Robins et al., 2012). In addition, social robots used as avatars by healthcare professionals enable immersive telepresence, characterized by human-like contact and interaction capabilities and advanced communication features that are difficult to achieve using more traditional tools, such as environmental cameras alone (Schwarz et al., 2021).

Although the integration of social robots in clinical settings is expanding, existing literature predominantly focuses on fully autonomous or entertainment-oriented robots deployed for general pediatric distraction, anxiety reduction, or structured therapy. However, a critical research gap remains: there is a dearth of studies investigating the use of teleoperated humanoid avatars specifically designed for naturalistic clinical observation in highly complex, unstructured settings, particularly within child neuropsychiatry.

Compared with fixed environmental cameras, a mobile telepresence platform may offer a more flexible and embodied viewpoint, while also enabling clinicians to communicate and physically interact with people and objects in the environment rather than merely observing them through a camera. However, its added value for behavioral observation remains to be evaluated in clinical practice. Teleoperating a humanoid avatar within a waiting room introduces relevant Human-Robot Interaction (HRI) challenges, including the need for usable and intuitive interfaces for non-expert clinical operators, adequate visual feedback for remote observation, and safe physical interaction supported by compliant hardware.

To address this gap, this study provides a preliminary evaluation of the usability, perceived cognitive workload, and acceptability of the Alter-Ego platform from the perspective of clinical and technical professionals. Designed to offer a mobile, first-person vantage point for observing spontaneous behaviors in a child neuropsychiatry waiting room, the platform was evaluated in a simulated setting without pediatric patients as an essential first step before clinical deployment.

## 2 Methods

### 2.1 Participants

A total of 13 clinical and technical professionals (mean age = 29.6; SD = 7.1) from IRCCS Fondazione Stella Maris (FSM) participated in the study. Involved participants were among healthcare professionals working at a multidisciplinary research laboratory dedicated to the development and clinical translation of robotic and biomechatronic technologies. Participation was voluntary and limited to the evaluation of the robotic system in a simulated setting without the involvement of patients or the collection of sensitive clinical data. The sample was divided into two groups. The first group included seven clinicians (1 male) with heterogeneous professional backgrounds, such as psychologists, neuropsychomotor therapists of developmental age, and child neuropsychiatrists. The second group included six professionals with a more technical background (1 male), including biomedical engineers and biotechnologists, who routinely use technology within a clinical setting for children. This composition allowed us to gather different perspectives: on the one hand, a more clinical perspective focused on assessment, rehabilitation, and child development; on the other hand, a more technical perspective related to the practical use, implementation, and integration of technology in hospital-based clinical practice. The clinical and technical professionals were directly involved in the teleoperation of the Alter-Ego robot during the experimental sessions. Participation in the study was voluntary.

### 2.2 Study design

The study employed the Alter-Ego humanoid robot, developed by the Department of Information Engineering of the University of Pisa and the Italian Institute of Technology in Genoa. Before the experimental sessions, all participants received a 15-min training session. During this phase, they were introduced to the Alter-Ego robotic platform and instructed on the use of the teleoperation system, including the VR Oculus interface and the joystick controller. Following the training session, clinical and technical professionals teleoperated the robot while performing a set of predefined interaction tasks designed to simulate potential activities within the waiting room of a child neuropsychiatry clinic. As a preliminary usability evaluation, the study was conducted without pediatric patients and focused on the operators’ experience before future testing with the target population. The tasks were carried out in pairs: one participant teleoperated the robot, while the other took on the role of the child interacting with it. The participant simulating the child interacted naturally with the robot by responding to its verbal prompts and performing the actions requested by the robot during the interaction. This paired setup allowed participants to experience both the teleoperator role and the role of the interaction partner, while providing a controlled simulation of the intended interaction scenario. The operator controlled the robot through a VR Oculus headset, which provided a first-person perspective from the robot by displaying the video streams captured by the two cameras positioned in the robot’s eyes.

The tasks were selected to cover complementary functions potentially relevant to the intended scenario, including mobile observation, verbal interaction, and basic physical interaction with people and objects. The experimental tasks were the following:

- Exploration and interaction task: participants were asked to move the robot from point A to point B toward a person, explore the surrounding environment, and engage in a conversation through the robot.
- Delivery task: participants were required to pick up an object at point A using the robot’s hands, transport it toward a person located at point B, and deliver it.

Each task lasted approximately 10 min, and the order of task execution was randomized across participants to reduce potential order effects.

The experimental sessions took place in a dedicated area within FSM, configured to reproduce a possible waiting-room scenario.

To evaluate the usability of the robotic platform, participants completed the System Usability Scale (SUS) questionnaire after the experimental session. In addition, user fatigue and perceived mental workload were assessed through the NASA Task Load Index (NASA-TLX). Subjective perceptions of the robotic platform, including anthropomorphism, animacy, likeability, perceived intelligence, and perceived safety, were assessed using the Godspeed Questionnaire Series.

### 2.3 Questionnaires overview

The participants were asked to complete three standardized questionnaires immediately after using the platform. These instruments enable a rigorous evaluation of the perceived workload and mental fatigue, the usability of the robotic platform, and the perception of the robot’s features. Hence, we chose the NASA-TLX (Georgsson, 2020), the System Usability Scale (SUS) (Brooke, 1996), and the Godspeed Questionnaire Series (GQS) (Bartneck, 2023) questionnaires.

Additionally, to capture contextual insights not covered by the standardized instruments, three *ad hoc* supplementary items were developed specifically for this study:

- “How familiar are you with the virtual reality systems and/or gaming?”
- “During the time when you were teleoperating the robot, did you feel that you could interact with the person you were talking to as you would like (communication, physical presence, etc.)?”
- “How suitable do you consider the robot for interaction with a child?”

These questions' purpose was to better understand if a participant’s prior experience with VR or gaming influenced their subjective perception and provide the level of the quality of HRI from both sides: the operator’s and the interaction partner’s point of view.

It should be noted that these supplementary items are non-validated and were developed exclusively for exploratory purposes. Consequently, their results are interpreted purely as descriptive context to complement, rather than substitute for, the findings obtained from the standardized questionnaires.

### 2.4 Technical description of Alter-Ego robots

Alter-Ego (Lentini et al., 2019) is a two-wheeled humanoid robotic platform designed to bridge the gap between teleoperated avatars and fully autonomous agents presented on the left-hand side of Figure 1. Aligning with the need for approaches in assistive and social robotics, its architecture is modeled on semi-anthropomorphic morphology. From an HRI perspective (Riek, 2012, 119–136), this design choice serves a dual purpose: it facilitates seamless integration into human-shared environments and enhances social acceptance, which is particularly critical in pediatric and clinical contexts.

**FIGURE 1:**
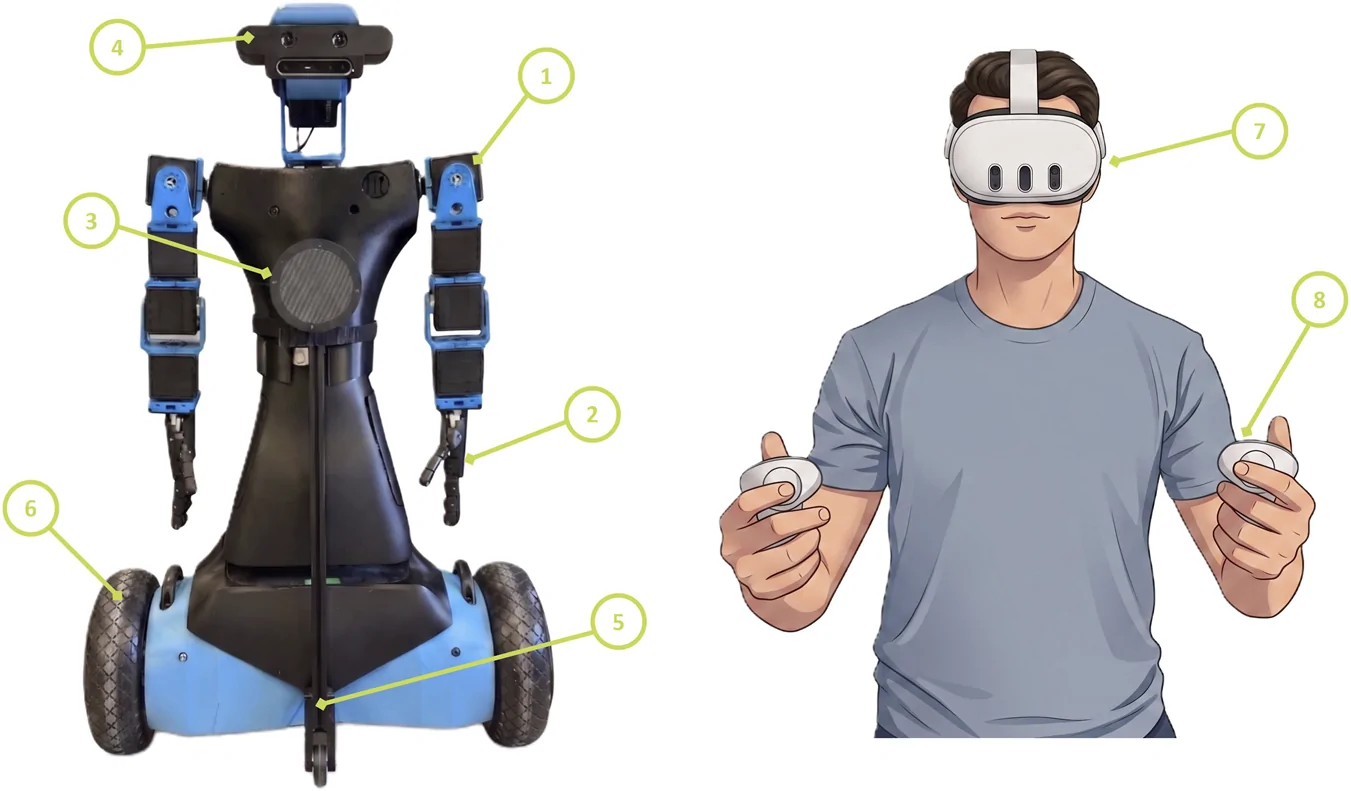
Overview of the experimental setup (left) the Alter-Ego humanoid robotic platform with highlights of its main technical features, including the Variable Stiffness Actuators (VSA Cubes, 1), the Pisa/IIT SoftHands (2), a Jabra microphone (3), a ZED camera (4), an integrated safety system (5), and the wheeled base (6) (right) the teleoperation pilot station showing an AI-generated illustration of a human operator equipped with a VR Meta Quest headset (7) and relative joystick controllers (8).

To ensure intrinsic safety during physical interactions, a critical requirement when operating around children or in healthcare scenarios, Alter-Ego’s upper body is equipped with modular Variable Stiffness Actuators (VSAs) (Catalano et al., 2011). These actuators replicate the agonistic-antagonistic behavior of human muscles, allowing simultaneous modulation of position and stiffness. This bio-inspired compliance ensures that the robot can perform accurate movements while remaining safe and resilient against unexpected impacts.

The use of the Pisa/IIT SoftHands (Catalano et al., 2016) simplifies grasp control by allowing adaptive grasping through a single command. The platform features two Pisa/IIT SoftHands, which exploit human-like structural synergies through a tendon-driven mechanism. This adaptive grasp allows it to handle objects of diverse shapes and sizes using a single command. This is translated to a single joystick command, useful for intuitive teleoperation with non-expert users.

The stereoscopic camera provides the operator with a first-person visual representation of the remote environment through the VR headset. Visual feedback is provided by a ZED-Mini stereoscopic camera mounted on a 2-DoFs (Pan/Tilt) VSA neck, which streams first-person video to a Meta Quest VR Headset, presented on the right-hand side of Figure 1. This high-fidelity immersive loop allows the teleoperator to naturally perceive the environment and observe the remote environment from the robot’s viewpoint.

Human-Robot engagement is further augmented by expressive facial features, including an RGB LED matrix mouth and mobile eyebrows capable of displaying primary emotional states (e.g., happiness, sadness, anger). The user can select an expressed emotion to dynamically adapt the interaction.

Verbal communication is supported by an integrated microphone and audio system, operating either *via* autonomous Large Language Model (LLM) frameworks (incorporating Speech-to-Text and Text-to-Speech pipelines) or through direct teleoperated audio streaming.

Mobility is managed by a two-wheel inverted pendulum mobile base that ensures agile navigation in unstructured environments. Crucially for non-expert clinical users, the platform autonomously stabilizes its vertical equilibrium (Zambella et al., 2019), allowing the operator to control linear and angular velocities *via* joystick levers without managing the robot’s balance. Onboard LiDAR enables simultaneous localization and mapping (SLAM) alongside automatic obstacle avoidance, ensuring safe navigation.

This combination of an intuitive control interface, embodied presence, and safe actuation makes Alter-Ego a potentially suitable platform for preliminary evaluation in clinical scenarios.

The versatility of Alter-Ego has been previously validated across diverse technical and social scenarios, including telepresence competitions (Zambella et al., 2025), contactless hospital support during COVID-19 (Fossati et al., 2020), public interactive guide applications (Garello et al., 2025), and pediatric performance art (Park et al., 2026).

## 3 Results

### 3.1 Results

In this section, we present the results of the questionnaire evaluation based on the collected data. The presented results refer to the overall activity described in the experimental setup, without splitting the data by individual task.

As a preliminary step, an evaluation of system usability, perceived workload, and user perception was conducted by comparing the two distinct participant groups: Clinicians (n = 7) and Technician Staff (n = 6). Given the small overall sample size (n = 13), subgroup comparisons and correlation analyses were assessed using the non-parametric Mann–Whitney U test for exploratory purposes only, and findings were interpreted descriptively.

The SUS ratings indicated acceptable usability for both groups (Figures 2C,D). Clinicians reported a slightly higher overall mean score of 73.9, with individual scores from 60.0 to 95.0, compared to the Technician Staff’s mean of 68.3, with a wider variation from 47.5 to 87.5. However, no significant differences emerged between the two groups (Mann–Whitney U = 16.5, p = 0.56). The associated effect size was small (rank-biserial correlation, r_rb_ = −0.20), indicating only minor differences between the two groups.

**FIGURE 2:**
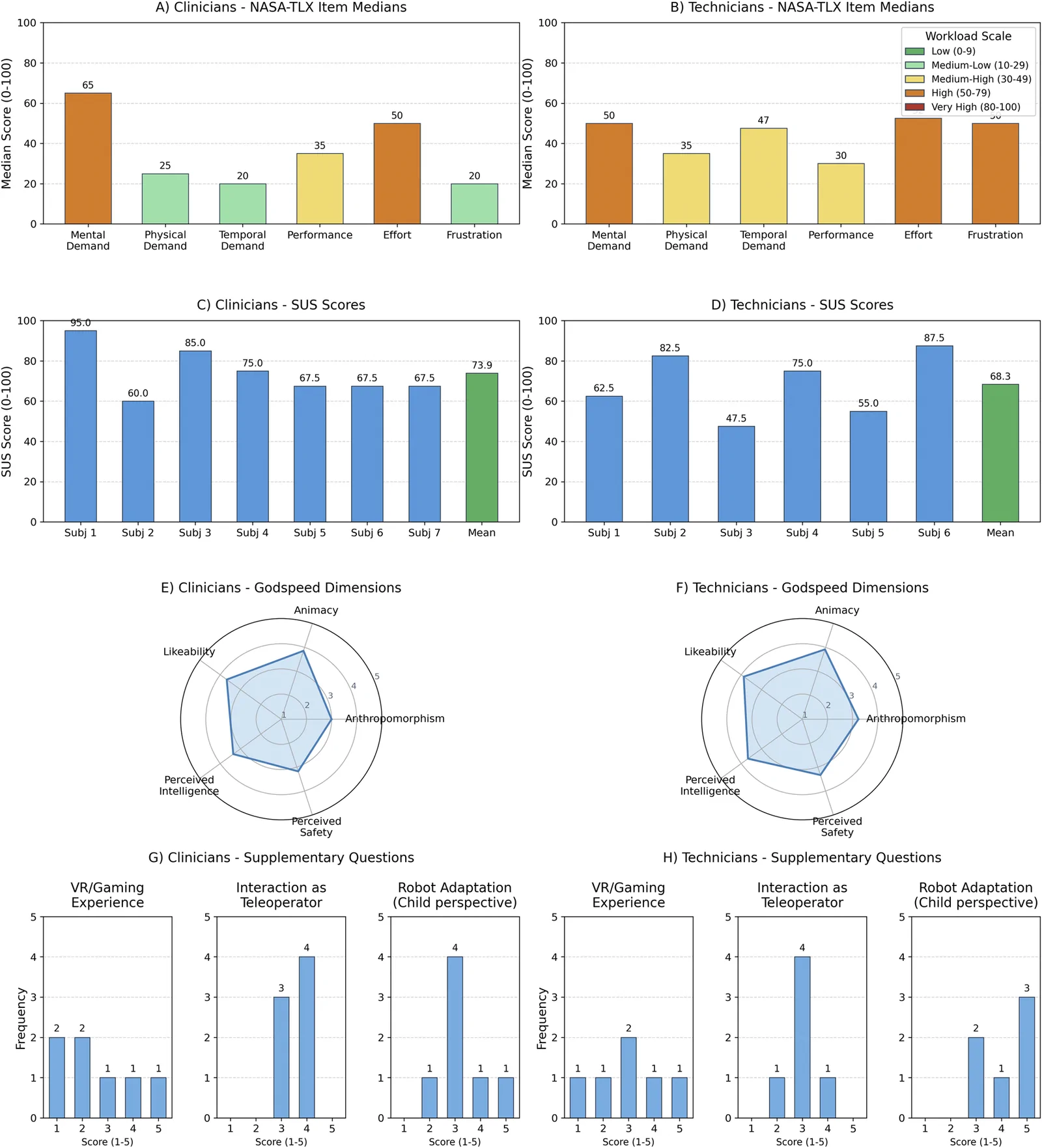
Evaluation results for the clinician and technician group. **(A,B)** Perceived workload dimensions from the NASA-TLX; **(C,D)** usability scores from the System Usability Scale (SUS); **(E,F)** robot perception profiles from the Godspeed Questionnaire Series; and **(G,H)** responses to the supplementary questions regarding prior experience.

Regarding perceived workload, the NASA-TLX of the two groups exhibited distinctly different yet quantitatively comparable profiles (Figures 2A,B). For clinicians, the most demanding dimensions were Mental Demand and Effort, both falling into the High workload band with median scores of approximately 65 and 50, respectively. However, when it came to frustration, the levels remained low, with the majority (57.1%) reporting Medium-Low frustration. On the other hand, the Technician Staff reported a generally higher workload across all dimensions evaluated. Notably, the majority of technicians (66.7%) reported High Mental Demand, High Effort, and High Frustration, with median scores for all three of these dimensions clustering in the High workload band (between 50 and 52). The most significant differences were observed only for Temporal Demand (Mann–Whitney U = 5.5, p = 0.03, r_rb_ = 0.74) and Frustration (Mann–Whitney U = 3.5, p = 0.013, r_rb_ = 0.83), with technical participants reporting higher scores than clinicians on both subscales.

From the Godspeed Questionnaire, the user perception of the platform was evaluated (Figures 2E,F). Both groups rated the robot most favorably in the Animacy and Likeability categories. These two categories received median scores of 4.0 and 3.75 among Clinicians, respectively, while Perceived Intelligence and Perceived Safety obtained moderate median scores of 3.25. The Technician Staff provided similar positive perception ratings, with median scores of 4.0 on Likeability, 3.9 on Animacy, and 3.9 on Perceived Intelligence, although the latter showed a wide spread of individual variance ranging from 2.25 to 5.0. Among these categories, both groups showed that Anthropomorphism received the lowest evaluations, yielding median scores of 3.0 for Clinicians and 2.8 for Technicians, indicating that the platform was largely perceived as machine-like rather than human-like. Comparisons between the two groups revealed no significant differences across any of the Godspeed subscales (all p > 0.05).

Regarding the remaining exploratory supplementary items (Figures 2G,H), both groups reported positive perceptions of interaction quality while teleoperating, with Clinicians reporting a mode score of 4.0 and Technicians a mode score of 3.0. Furthermore, both Clinicians and Technicians evaluated the platform’s suitability for interacting with children favorably, yielding mode scores of 3.0 and 5.0, respectively.

Following this detailed subgroup comparison, data were further aggregated to evaluate the overall trends across all participants (Figure 3).

**FIGURE 3:**
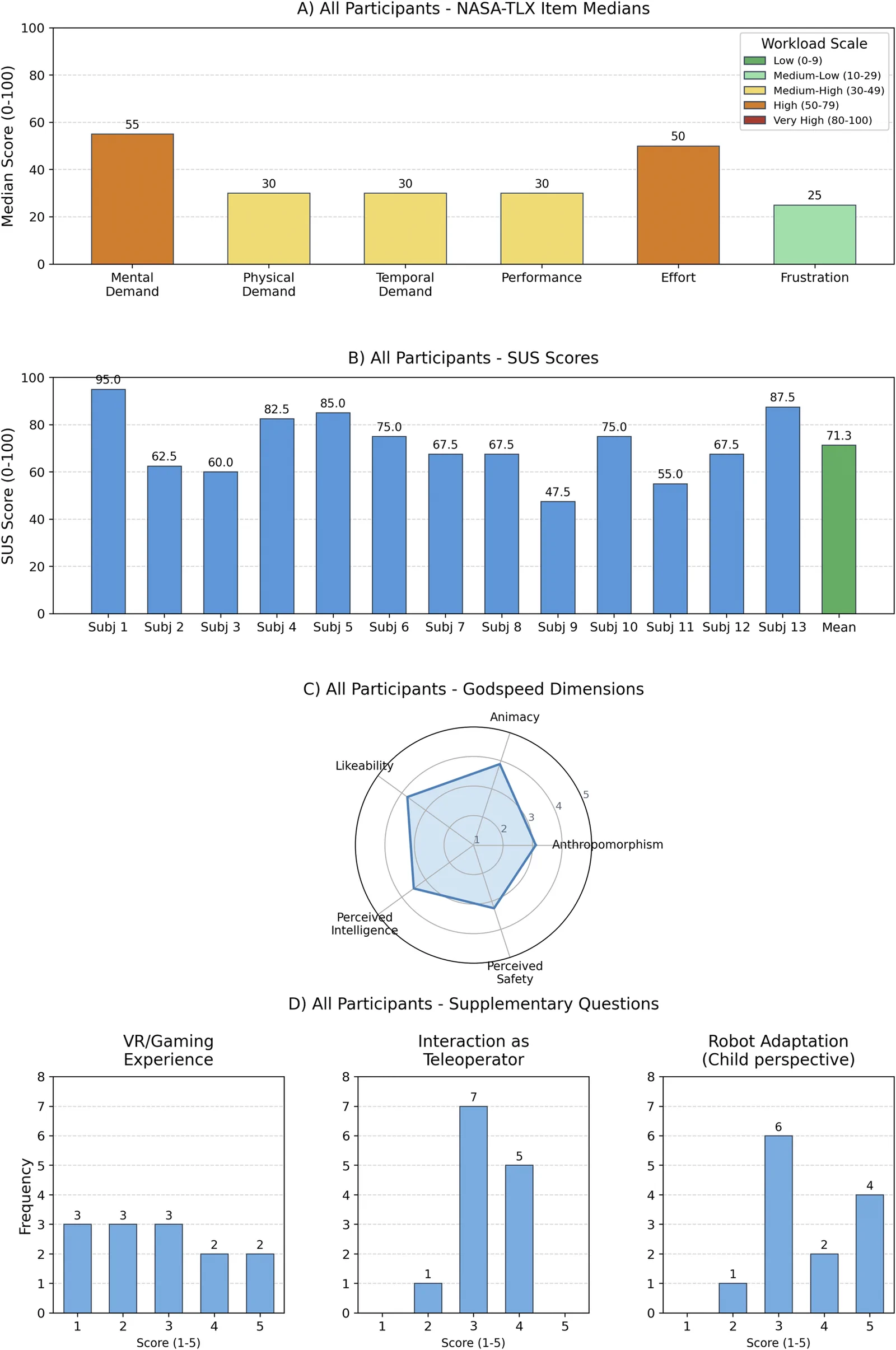
Evaluation results for the overall sample. **(A)** Perceived workload dimensions from the NASA-TLX; **(B)** usability scores from the System Usability Scale (SUS); **(C)** robot perception profiles from the Godspeed Questionnaire Series; and **(D)** responses to the supplementary questions regarding prior experience.

The NASA-TLX analysis showed that teleoperation required mainly cognitive and effort loads (Figure 3A). Mental Demand and Effort had the greatest share in the total workload score, and the medians for these scales lay in the high workload spectrum (50–79). This interpretation is also supported by the percentage distribution showing that 69% and 61% of participants felt a high or very high level of mental and effort demand, respectively. In turn, Frustration received the lowest median score among the NASA-TLX dimensions (10–29).

The total SUS score was 71.3 points, exceeding the commonly accepted usability score of 68 and suggesting an acceptable usability level (Figure 3B). At the same time, individual scores demonstrated some variations between users, between 47.5 and 95.0 points.

Overall, the Godspeed Questionnaire demonstrated a positive evaluation of the robot (Figure 3C). The two top scores in the questionnaire belonged to Animacy and Likeability, both having medians equal to 4.0 points. Perceived Intelligence varied greatly among the participants (2.25–5.0), which could suggest that personal interactions with the robot played an important role here. Medians of Perceived Intelligence and Safety showed moderate results of 3.25 and 3.0 points, respectively.

In general, no significant correlations were found between familiarity with VR technologies or gaming experience and any of the outcome measures considered in the study, including SUS, NASA-TLX, and Godspeed scores (Figure 3D; all p > 0.05).

Across the entire sample (Figure 3D), responses to the *ad hoc* supplementary items indicated that participants felt generally capable of interacting as desired during teleoperation (92.3% scoring ≥3) and perceived the robot as suitable for child interaction (92.3% scoring ≥ 3).

A comprehensive numerical and statistical summary of these data is provided in Figure 4.

**FIGURE 4:**
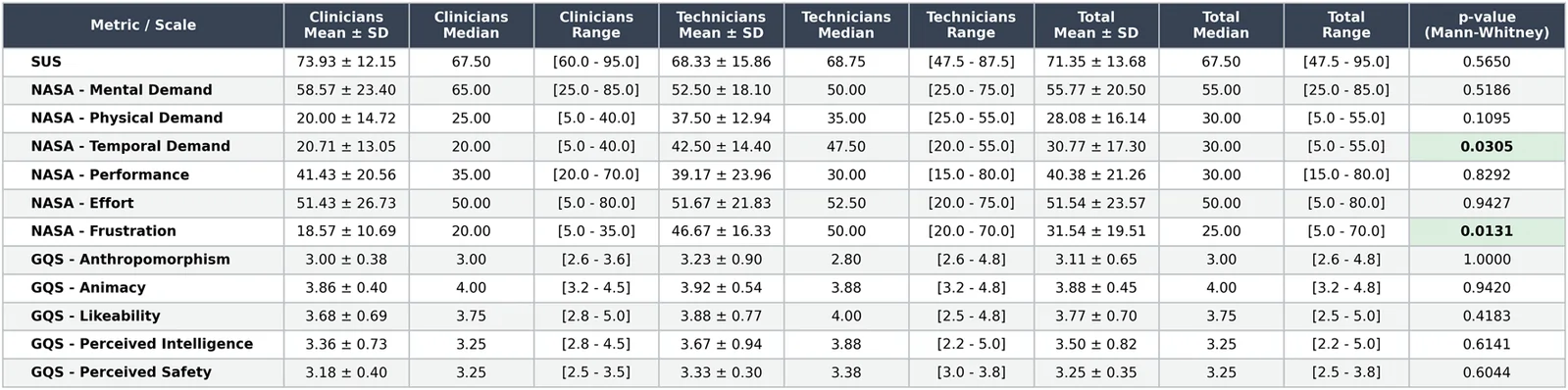
Summary of the quantitative results for the System Usability Scale (SUS), NASA Task Load Index (NASA-TLX), and Godspeed Questionnaire Series (GQS). Data are reported as Mean and Standard Deviation (SD), Median, and Range for Clinicians (n = 7), Technician Staff (n = 6), and the aggregated total sample (n = 13). Statistically significant differences between groups (p < 0.05) are highlighted in green, evaluated *via* non-parametric (Mann-Whitney U) tests.

### 3.2 Discussion

This preliminary study is part of the body of literature dedicated to making clinical settings increasingly welcoming and child-friendly, with the aim of improving the healthcare experience. At the same time, the study arises from the clinical need to observe and assess children’s behavior in more ecological and less structured contexts compared to traditional clinical settings. For this purpose, the Alter-Ego robotic platform was evaluated by clinical and technical professionals involved in the teleoperation, with the long-term aim of facilitating its implementation in the waiting room of a child neuropsychiatry clinic. Given the limited sample size, the statistical analyses, particularly subgroup comparisons and correlation analyses, should be considered as exploratory results. Overall, the findings provide encouraging evidence regarding the feasibility of using the platform in clinical settings and offer valuable insights into the user experience associated with robot teleoperation.

The results of the SUS questionnaire indicated an overall usability score above the commonly accepted threshold of 68, suggesting that the system is generally perceived as usable and acceptable by potential operators. Although individual scores showed some variability, the average SUS score supports the notion that clinical and technical professionals were able to effectively interact with and control the platform following a relatively brief training session. Both groups were able to complete the experimental activities and reported broadly comparable usability scores, although the small sample does not allow firm conclusions regarding differences between professional backgrounds.

The workload assessment revealed a more nuanced picture. Across participants, Mental Demand and Effort emerged as the main contributors to the teleoperation experience, indicating that controlling the robot requires sustained attention and cognitive engagement. This finding is not unexpected, considering that participants were exposed to the platform for the first time and received only a short training session before performing the tasks. Despite the relatively high cognitive demands, Frustration levels remained generally low, suggesting that participants perceived the interaction as manageable and did not experience substantial negative emotional responses during teleoperation. Interestingly, technical participants reported significantly higher Temporal Demand and Frustration compared to clinicians. One possible explanation is that technical users may have been more sensitive to the limitations of the system or may have developed higher performance expectations regarding responsiveness and control precision. Clinicians, on the other hand, may have focused more on the interactional and communicative aspects of the tasks rather than on technical performance. Although these interpretations remain speculative, they suggest that professional background may influence how users evaluate specific aspects of the teleoperation experience.

The Godspeed results further support the acceptability of the platform. Participants generally perceived the robot as animated, responsive, and likeable, while ratings of perceived safety were also positive. These characteristics are particularly relevant in pediatric healthcare environments, where engagement, trust, and positive emotional responses are essential for successful interactions. At the same time, anthropomorphism received the lowest ratings among all dimensions. Rather than representing a limitation, this finding may indicate that users clearly recognized the robot as a technological tool while still attributing positive social qualities to it. This balance may be advantageous in clinical contexts, where excessive anthropomorphism is not necessarily required to foster engagement and communication.

In line with these findings, the exploratory supplementary items revealed that clinical and technical professionals felt able to maintain satisfactory remote communication during teleoperation and perceived the Alter-Ego platform as well-suited for interaction with children, supporting its potential acceptance in pediatric healthcare scenarios. An additional finding of interest is the absence of significant correlations between familiarity with VR technologies or gaming experience and any of the questionnaire outcomes. Within this sample, no clear association emerged between prior VR or gaming experience and the questionnaire outcomes. From a clinical implementation perspective, this result could suggest that the platform may be accessible to a broad range of clinical and technical professionals regardless of their technological background. However, given the limited sample size, these findings should be interpreted with caution and do not exclude a possible influence of previous technological experience.

Taken together, these findings provide preliminary information limited strictly to clinical and technical professionals' experience - specifically system usability, perceived cognitive workload, and acceptability - in a simulated environment.

Even with the exploratory nature of the study, it highlights favorable feedback from healthcare professionals regarding system interaction and usability, supporting and encouraging further investigation of the Alter-Ego platform in more realistic clinical workflows.

The results also highlight the importance of interdisciplinary collaboration between clinicians and roboticists in the design and evaluation of robotic technologies intended for healthcare settings.

### 3.3 Limitations

This preliminary study has some limitations. First, the relatively small and young sample size limits the statistical power of the analyses and the generalizability of the results. Future studies should therefore include larger and more heterogeneous samples. Second, although the proposed activities were designed to replicate realistic clinical scenarios, how children will interact with the robot and how its use might influence their behavior in a real-world setting such as a waiting room remains unclear. Finally, although no significant discomfort was reported during the experimental sessions, the prolonged use of the immersive VR interface required for teleoperation could be fatiguing for some operators. This potential limitation could be mitigated through a training period, allowing users to progressively adapt to the teleoperation system before its routine clinical use.

## 4 Conclusion

This study explored the usability, perceived workload, and acceptability of the Alter-Ego humanoid robot as a telepresence platform intended for future use in child neuropsychiatry waiting rooms. Conducted without the involvement of pediatric patients, the study provided preliminary feedback from clinical and technical professionals with different clinical and technical backgrounds.

The findings suggest that the platform may be usable after brief training and was generally positively perceived by participants, although teleoperation required a relevant degree of mental effort and individual usability scores varied. These results should therefore be interpreted as an initial operator-centered evaluation rather than as evidence of clinical effectiveness.

The study is limited by the small sample size, the simulated setting, and the absence of children and caregivers. In addition, it did not assess the quality or clinical relevance of the observations obtained through the robot. Future studies should evaluate the platform in real clinical workflows, involving pediatric patients and caregivers, and examine both user acceptability and the extent to which robot-mediated observation can provide meaningful information to healthcare professionals.

## Data Availability

The original contributions presented in the study are included in the article/supplementary material, further inquiries can be directed to the corresponding author.
